# Work situation and professional role for midwives at a labour ward pre and post implementation of a midwifery model of care – A mixed method study

**DOI:** 10.1080/17482631.2020.1848025

**Published:** 2020-12-01

**Authors:** Malin Hansson, Ingela Lundgren, Anna Dencker, Charles Taft, Gunnel Hensing

**Affiliations:** a Institute of Health and Care Sciences, Sahlgrenska Academy, Gothenburg University, Gothenburg, Sweden; b Department of Public Health and Community Medicine, Institute of Medicine, Sahlgrenska Academy, Gothenburg University, Gothenburg, Sweden

**Keywords:** Midwifery, mixed method, work situation, professional role, stress, burnout, demand and control

## Abstract

**Purpose**: To explore and analyse the experience of work situation and professional role for midwives at a labour ward pre and post the implementation of a midwifery model of care (MiMo).

**Methods**: A simultaneous mixed method was used. The qualitative core component departed from three focus group interviews (n = 16 midwives). Secondary inductive and deductive content analysis was performed using an unconstrained matrix to make a corresponding comparison of the different time points. The supplemental component was a quantitative survey about the work situation (n = 58).

**Results**: The qualitative results pre the implementation showed three categories: *Balance between Women and Organization, Midwives—Diverse as both Profession and Person*, and *Strained Work Situation*. Post the intervention they transformed to *Balance between Midwifery and Organization, Midwives—An Adaptable Profession, Strained Work Situation*, and a new category *Ability to concretize midwifery* was found. There were no significant differences in the measures of work situation in the quantitative analyses.

**Conclusions**: The synthesized findings based on the qualitative part show that MiMo has a potential to strengthen the professional role and midwifery practice. As such, MiMo has the capability to offer benefits to the labour wards with additional considerations.

## Introduction

Midwives’ work situation and professional role are important for maternity care. A review of midwife-led continuity models in care for childbearing women revealed fewer interventions compared to other care models, higher satisfaction among the women, and at least comparable adverse outcomes for the women and their infants in relation to women receiving other models of care (Sandall et al., 2016). There were also benefits for midwives, including improved job satisfaction and less occupational burnout (Homer, 2016). However, most midwives do not work with continuity models of care, in high-income countries due to a medico-technical approach (Davis-Floyd, 2001) related to discontinuity for women during childbearing. High levels of work-related stress, burnout, and heavy workload in midwives at labour wards have been reported from countries such as Ireland, Sweden, Denmark, and Norway (Borritz et al., 2006; Henriksen & Lukasse, 2016; Hildingsson et al., 2013; Mackin & Sinclair, 1998). A recent qualitative study from Australia states that midwives are fighting a losing battle trying to cope with unbearable levels of work-related stress, a situation described as “war like”, that resulted in midwives considering leaving the profession (Geraghty et al., 2019). Midwives’ professional identity is according to (Zhang et al., 2015) a struggle and a negotiation between the external medical model (obstetrical nurse) and the internal definition (professional midwife) connected to the philosophy of normal birth. This bisect view of midwifery creates a dissonance in the midwives professional identity, which can contribute to midwifery care in hospitals being more difficult. The results correspond with UK studies that reported that midwives are often dissatisfied with the standard of provided maternity care as well as their professional role (Curtis et al., 2006; Finlayson et al., 2002; “Why midwives leave,” , 2005).

There is an increasing focus on risks in society in general and in health care in particular. Risk-centred policies may constrain women-centred care and facilitate the medical approach (Leap, 2009; MacKenzie Bryers & van Teijlingen, 2010). Internationally the risk-focused medical-technical model of care is dominant in labour wards, leading to a large rate of interventions even in normal births (Ólöf Ásta, 2011). However, in recent years, women-centred care has gained interest both in research and in developing guidelines. Women-centred care is interrelated to the salutogenic approach and is the main goal in midwifery as well as a theoretical perspective (Bryar & Sinclair, 2011; Magistretti et al., 2016). The characteristics of women-centred care are partnership, holism, respect, and safety. The consequences of this kind of care are empowerment of the women, an autonomy for the care provider, and also a societal reform (Horiuchi et al., 2006). Women-centred care also shift the locus of control from the institution and professionals towards the woman (Fahy, 2012; Leap, 2009). An ambition in midwifery work is that midwives should recognize each individual woman’s physical, emotional, social, spiritual and cultural needs, expectations, and context. All this should be defined by the woman herself, not by the caregiver (Fahy, 2012; Hunter, 2016).

**Figure 1.**
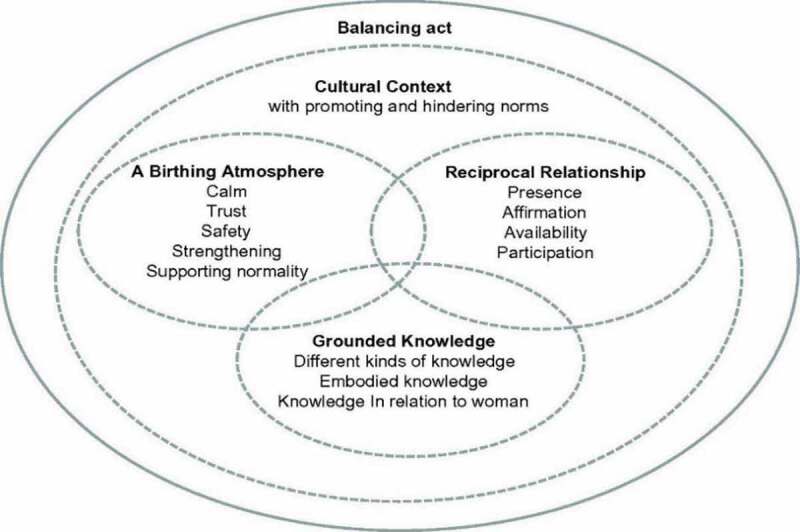
Midwifery model of women-centred childbirth care developed in Swedish and Islandic settings, Berg et al. (2012), page 83 (Berg et al., 2012)

As stated above, woman-centred care is an important aspect of midwives’ professional role (Berg et al., 2012; Bryar & Sinclair, 2011), conceptualized theoretically in midwifery models of care focusing on the midwife–woman relationships and how to support normal birth (Bryar & Sinclair, 2011). There are some theoretical midwifery models of care but few have been evaluated (Lundgren et al., 2020). Further, since the organization of care and professional role for midwives differ in an international perspective, a Midwifery Model of Woman-Centred Care (MiMo) was developed based on the Nordic context, Sweden, and Iceland (Berg et al., 2012). The model includes five main themes (Figure 1). The midwife is together with the woman, using grounded knowledge, forming a reciprocal relationship to create a birthing atmosphere. The three central dimensions are conducted by the midwife through a balancing act in a cultural context that comprises both promoting and hindering norms for conducting woman-centred care (Berg et al., 2012).

The MiMo research project has an overall aim to evaluate and assess the usefulness of MiMo in practice and its impact on the outcome of childbirth care, including developing guidelines for using the model in maternity care.

To our knowledge, no previous studies have explored the implementation of midwifery models of care in relation to midwives work situation. The aim of this study was to explore and analyse the experience of work situation and professional role for midwives at a labour ward pre and post the implementation of MiMo.

## Materials and methods

### Design

The study presented here is a part of the MiMo research project but has its own focus about the work situation and professional role for midwives at a labour ward. This study has a simultaneous mixed-method design with a focus on the qualitative core component and uses the quantitative data as a supplement component (Morse & Niehaus, 2009). The decision to use mixed method was based on the complex research questions and the aim to explore both the micro- (e.g., individual) level and the meso- (e.g., group) level (Morse, 2016). A secondary analysis was used on the rich MiMo interviews (Lundgren et al., 2020) in this study, to maximize data utility and to apply a work situation and professional role focus on existing data (Heaton, 2004).

In the MiMo research project, an intervention was implemented at two wards at a university hospital in the western part of Sweden. One was used for the intervention and one as control. Additionally, a study was carried out in Iceland to develop guidelines for practice. A qualitative study evaluated the usefulness in practice by focus group interviews with different health professionals at the intervention ward (Lundgren et al., 2020). Focus group interviews with midwives pre and post the intervention were analysed in this study together with original survey data about the work situation pre and the post intervention.

In the study presented here, the work situation was analysed before and after the implementation of MiMo. The theoretical MiMo was implemented in the existing care as an approach to midwifery and woman entered care. Pre the intervention, all midwives at the intervention ward were invited to one 8-h day of education in January 2015. The education consisted of a presentation of the model together with handouts, including a MiMo card with the model of the MiMo research project to be used for guiding the intervention on the ward. Group discussions based on the participants’ own questions related to the model were conducted. The model was also presented for obstetricians, assistant nurses, and managers working at the labour ward. To continue developing knowledge and experience with the model, the midwives at the intervention ward attended reflection groups led by one of the researchers and four MiMo midwives. The intervention was performed between March 2015 and March 2016.

Midwives were interviewed pre and post the intervention in focus group interviews (Table I). After receiving a brief explanation about MiMo (Figure 1), two opening questions were asked both pre- and post-intervention: “What is your opinion of the applicability of a midwifery model of woman-centred care?” and “What is your professional role related to woman-centred care?”. The last question about the professional role is closely related to the work situation. In the study presented here, the focus group interviews were reanalysed in a secondary analysis focusing midwives work situation and professional role (Heaton, 2004). The quantitative supplement component consists of a survey designed for the present study, further described below.

**Table I.** Characteristics of participants in the three focus group interviews, Sweden 2015–16

Focus group interview (no.)	Midwives (n)	Years of work experience at labour ward, range
Pre-intervention (1)	5	2–16
Post-intervention (2) Post-intervention (3)	7 4	2–33 7-17

**Figure 2.**
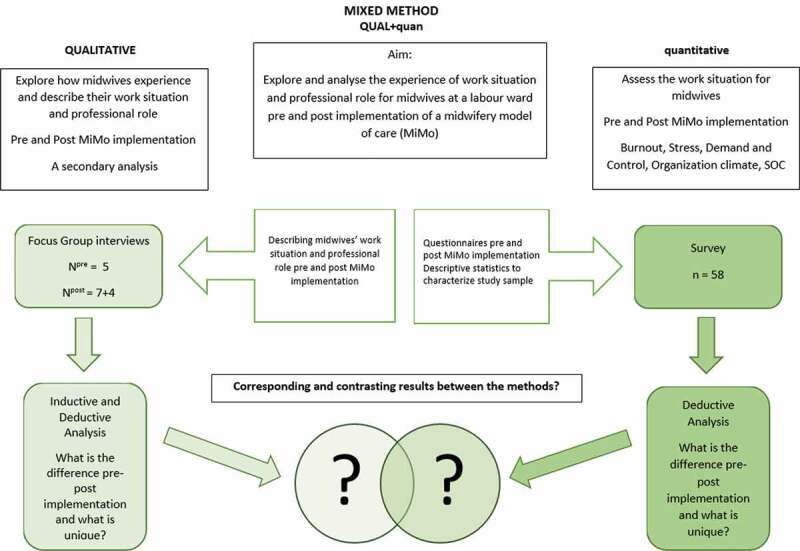
Study design and aim of the (QUAL + quan) mixed-method sub-study of the MiMo-project, Sweden 2015–16

In this study, a simultaneous qualitative and quantitative (QUAL + quan) mixed-method approach (Morse & Niehaus, 2009) was used. QUAL + quan meaning an inductive-simultaneous data collection design where the core component is qualitative and drives the quantitative supplement component analysis (Morse & Niehaus, 2009; Schoonenboom & Johnson, 2017). The overall design and aim of this study are presented in Figure 2. The core component was a qualitative secondary analysis (Heaton, 2004) of data collected during the original focus group interviews in the MiMo project. The analysis explored how midwives experienced and described their work situation and professional role, pre and post the implementation of MiMo (Lundgren et al., 2020). The simultaneous gathering of the quantitative supplemental component comprised of an analysis of a longitudinal survey, measuring different aspects of the work situation for midwives pre and post the implementation of MiMo. The quantitative analysis was driven by the qualitative results—i.e., the survey was reviewed section by section to identify the items that had measurements corresponding to the qualitative categories. Some quantitative data were not corresponding to the qualitative categories, thus were not included in this study, for example, SF36.

### Context

The work situation was analysed pre and post the implementation of the theoretical MiMo at one low-risk labour ward in a large urban hospital in the southwest region of Sweden. The participating hospital has a ward for women with high-risk not involved in this study. The labour ward was for women with singleton uncomplicated pregnancies and expected uncomplicated births from gestational week 34 + 0. However, induction of labour was common as well as women with minor complications such as gestational diabetes and gestational hypertension. In addition, women with stillbirths received care on the ward. The ward cared for 4,570 birthing women in 2015 and 4,725 in 2016. Continuity of care is not offered in Sweden as pregnancy care is organized by primary healthcare midwives and not labour ward midwives at the hospital. One-to-one care is not provided at the labour ward as midwives often care for more than one woman at the same time.

### Data collection and participants

#### Qualitative core component

Focus group interviews were done pre and post the intervention with a total of 16 midwives (Table I). A purposive sampling was used, aiming for variation in work experience. The focus group interviews were held at the hospital for the convenience of the participants. None of the midwives participated in the focus groups both pre- and post-intervention. A secondary analysis (Heaton, 2004) of the three focus group interviews was conducted with the objective to explore how midwives experienced and described their work situation and professional role in relation to MiMo.

#### Quantitative supplemental component

A survey was answered by midwives before (n = 78) and post-intervention (n = 58). The original 78 midwives were a total sample at the participating ward. Ten of the original sample had either quit their job or were on sick or parental leave and ten did not answer the post-intervention survey. These 20 participants were excluded since we used a paired analysis (pre and post). Pre-intervention paper survey was distributed, answered, and submitted on-site at the end of the MiMo education day. At follow-up, a digitalized Webropol online survey tool was used to administer the survey. Midwives were contacted through their work email. The SPSS (Statistical Package for the Social Sciences) version 24 was used for the registration and analysis of the quantitative data. Descriptive statistics were used to characterize the study sample (Table II). Our focus was not on the absolute levels of the assessed measurements but rather on changes from pre- to post-intervention. To determine differences pre and post the intervention, Wilcoxon signed-rank tests were conducted.

**Table II.** Characteristics of participants in the survey of work situation of the MiMo project, Sweden 2015–16

	Midwives at labour wards (n = 58*)
	Mean (range)
Age in years	44 (27–65)
Number of children	2 (0–4)
Children living at home	2 (0–4)
Years working as a midwife	11 (1–33)
Years at current workplace	9 (1–32)
Working hours/week	33 (9–40)
	n (%)
Marital Status	
Single	8 (14)
Cohabiting or married	45 (77)
Couple living apart	4 (7)
Widowed	1 (2)
Education	
Bachelor’s degree	35 (60)
Master’s degree, one year	23 (40)
Employment	
Full-time	30 (52)
Part-time	28 (48)
Work	
Day time weekdays	2 (2)
Two shifts	38 (66)
Three shifts	9 (16)
Night shift	9 (16)

*The number of midwives that responded to the survey pre and post the intervention of MiMo was 58 out of the original 78 participants.

Measurements used were the Copenhagen Burnout Inventory (CBI), the Perceived Stress Scale (PSS), the job demand-control-support model, seven items from the Work Stress Questionnaire (WSQ) about perceived work stress and the Sense of Coherence Questionnaire (SOC). The Copenhagen Burnout Inventory (CBI) measures physical and psychological exhaustion with three sub-scales: Personal Burnout (six items), Work-Related Burnout (seven items), and Client-Related Burnout (six items). Scores of the 19 items range from 0 to 100 on each item (scoring 0, 25, 50, 75, or 100); higher scores indicate higher levels of burnout. The cut-off is 50. Scores 50–74 are moderate burnout, 75–99 are high burnout, and 100 are severe burnout (Kristensen et al., 2005).

Perceived Stress Scale (PSS) consists of 14 items and measures which situations are perceived as stressful on a 5-point Likert scale. The scale score ranges from 0 to 56. To obtain the total PSS score, the scores of the positive items (items 4, 5, 6, 7, 9, 10, and 13) are reversed and then summed with the scores of the remaining items. High scores indicate a high level of stress (Cohen et al., 1983). The PSS is not a diagnostic instrument, so there are no cut-offs.

In the five-item job demand scale (range 5–20), a high score indicates high demand. In the six-item job control scale (range 6–24), a high score indicates high control. Social support was measured by a scale ranging from 6 to 24, where a high score indicates high social support (Karasek, 1990). Questions from the work stress questionnaire (WSQ) were used (K. Holmgren, Hensing et al., 2009). Three items about organization climate and four items on work commitment were included in this survey that were applicable for a healthy and working population. A 4-point Likert scale is used in WSQ, and the reliability and face validity have been tested and found to be good (Holmgren, Dahlin-Ivanoff, et al., 2009).

The short version of the Sense of Coherence Questionnaire (SOC) measures comprehensibility, manageability, and meaningfulness. The SOC consists of 13 items answered on a 7-point Likert scale. The score range is from 13 to 91, and the total score is obtained by reversing items 1, 2, 3, 7, and 10. A high score indicates high levels of sense of coherence (Antonovsky, 1987). The cut-off score is 60. Less than 60 is low SOC, 61–75 is moderate SOC, and 76–91 is high SOC (Eriksson & Lindström, 2007).

Previous research has found that the psychometric properties, the validity, and the reliability of the surveys included were good (Cohen et al., 1983; Eriksson & Mittelmark, 2017; Holmgren, Dahlin-Ivanoff, et al., 2009; Karasek, 1990; Kristensen et al., 2005).

### Ethics

The study was approved by the Regional Ethical Reviewer Board in Gothenburg 2014 no. 840–14.

### Data analysis

#### Mixed method

The mixed method had an inductive theoretical drive with a qualitative core component and a simultaneous deductive quantitative supplementary component (Figure 2). The point of interface between the methods was in the results section, synthesizing the narrative (Morse, 2016). According to (Morse, 2016), the qualitative findings form the theoretical frame of the results, and the quantitative analysis is used to expand certain details of these results. The emerged theoretical QUAL model was used as a foundation and drove the quantitative analysis to develop the understanding of the qualitative result further as well as to analyse whether the results were corresponding or contrasting over time (Morse & Niehaus, 2009; Schoonenboom & Johnson, 2017).

#### Qualitative analysis

Data were analysed using inductive and deductive content analysis according to (Elo & Kyngäs, 2008). Thus to explore change in the experience of the work situation over time. The focus group interviews were transcribed verbatim and imported to NVivo (Qualitative Data Analysis Software; QSR International Pty. Ltd.) version 11, which was used as a sorting tool during the analysis of the text. The analysis was primarily performed by the first author (MH) but in constant comparison with the co-authors to ensure trustworthiness and credibility (Elo & Kyngäs, 2008).

The qualitative analysis was performed in two steps. First, an inductive analysis was performed in the focus group interview pre the intervention. Second, a deductive and in parts inductive unconstrained matrix analyses of the two focus group interviews post the intervention were conducted to make a corresponding comparison at the different time points (Elo & Kyngäs, 2008). The analyses started with making sense of the data as a whole then the open coding was conducted in NVivo to create coding sheets. Next, these were grouped into sub-categories. The sub-categories were classified under higher-order headings, and the main categories were classified by categorizing and abstracting the data (Elo & Kyngäs, 2008). Three generic categories were generated in the analysis at pre-intervention. These categories and sub-categories were used as an unconstrained matrix (Elo & Kyngäs, 2008) during the analysis of the post-intervention interviews, which resulted in four generic categories of which some were coherent with pre-intervention but others were diverse. Finally, a corresponding comparison was done between the different time points (Elo & Kyngäs, 2008).

#### Quantitative analysis

**Figure 3.**
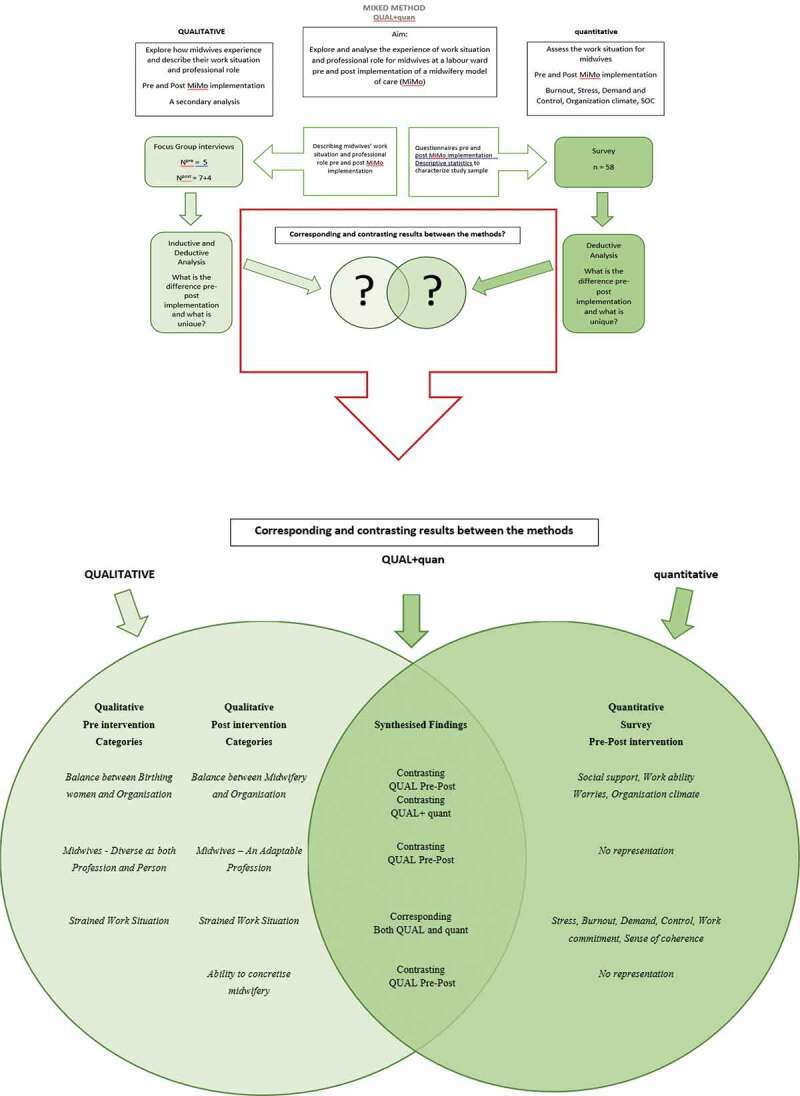
Point of interface between qualitative and quantitative (QUAL + quant) results and synthesized findings

The qualitative concepts that emerged (Table III) guided the choice of quantitative measurements to analyse of how the implementation of MiMo affected the midwives’ work situation. The survey was systematically reviewed, section by section, by two of the researchers to identify items that measured corresponding aspects as the identified qualitative categories. Items with corresponding aspects were analysed by Wilcoxon signed-rank test pre and post the MiMo, to expand the qualitative results (Morse, 2016). The quantitative analysis was consequently driven by the theoretical qualitative frame and core component. The supplementary quantitative results (Table IV) were synthesized with the qualitative core component findings (Figure 3).

**Table III.** Experiences of the professional role and work situation pre and post the MiMo intervention, Sweden 2015–16

Pre-intervention categories and sub-categories	Post-intervention categories and sub-categories
**Balance between Birthing Women and Organization** Adaptation to women’s experiences Controlling guidelines Labour—natural process not a medical event Teamwork The goal of being present and supportive	**Balance between Midwifery and Organization** Midwifery processes Birthing as a complex but natural process, dissolving medical focus Teamwork The goal of being present and supportive
**Midwives—Diverse as both Profession and Person** Midwives work differently Different personalities	**Midwives—An Adaptable Profession** Midwives customize their work Get to know oneself as a midwife
**Strained Work Situation** Lack of support for the supporter Lack of time Commitment and self-criticism	**Strained Work Situation** Lack of support for the supporter Lack of time Commitment and self-criticism
	**Ability to Concretize Midwifery** Professional reflection Communication midwife to midwife Having words for midwifery

**Table IV.** Work situation pre and post the intervention of MiMo assessed by survey measurements, MiMo project, Sweden 2015–16

Survey	Mean pre (SD) *n* = 58	Mean post (SD) *n* = 58	Median pre-post *n* = 58	*p* value*
**Social support** (range 6–24) **Work ability in relation to work demands**: Knowledge Mental Emotional Collaborate Physical **Worrying about**: Reorganization Technology Manage work Get unemployed Subjected to Bullying Subjected to Sex harassment **Organizational Climate**: Boss consider your opinions Conflict involvement Uneasiness going to work	20.77 (±2.19) 1.59 (± .59) 1.67 (± .69) 1.76 (± .66) 1.43 (± .53) 1.67 (± .66) 3.14 (±1.71) 2.16 (±1.44) 2.26 (±1.33) 1.11 (± .37) 1.36 (± .82) 1.02 (± .13) 1.66 (± .52) 3.45 (± .65) 2.00 (± .73)	19.98 (±2.36) 1.47 (± .54) 1.69 (± .71) 1.71 (± .71) 1.43 (± .57) 1.59 (± .62) 2.60 (±1.61) 1.98 (±1.26) 2.14 (±1.42) 1.07 (± .32) 1.36 (± .83) 1.03 (± .18) 1.86 (± .64) 3.53 (± .66) 2.11 (± .98)	21.00–20.00 2.00–1.00 2.00–2.00 2.00–2.00 1.00–1.00 2.00–2.00 3.00–2.00 2.00–2.00 2.00–2.00 1.00–1.00 1.00–1.00 1.00–1.00 2.00–2.00 4.00–4.00 2.00–2.00	.097 .264 .991 .553 .990 .474 .100 .594 .441 .429 .741 .564 .062 .424 .766
**Stress** (range 0–56) **Burnout** (range 0–100) Personal burnout Work-related burnout Client-related burnout **Demand** (range 5–20) **Control** (range 6–24) **Work commitment**: Engagement High demands on oneself Hard to say no Responsibility **Sense Of Coherence** (range 13–91)	19.16 (±7.05) 36.93 (±15.54) 32.37 (±13.33) 20.04 (±13.09) 15.48 (±1.86) 19.57 (±1.60) 1.55 (± .60) 1.14 (± 35) 1.93 (± .53) 2.19 (± .55) 74.12 (±8.54)	20.07 (± 6.25) 39.71 (±17.76) 34.98 (±15.63) 23.25 (±15.51) 15.78 (± 1.46) 19.41 (± 1.81) 1.64 (± .67) 1.19 (± .44) 1.95 (± .69) 2.02 (± .64) 74.88 (±10.56)	19.00–19.50 37.50–37.50 30.65–32.14 16.67–20.83 15.50–16.00 20.00–20.00 1.50–2.00 1.00–1.00 2.00–2.00 2.00–2.00 75.00–78.00	.288 .369 .329 .153 .334 .839 .455 .405 .861 .077 .272

* Wilcoxon signed-rank test pre and post the MiMo.

## Results

### Qualitative core component result

Three categories were identified in the pre-intervention focus group interviews and four categories were identified in the post-intervention focus group interviews from the corresponding comparison between the different time points (Table III).

#### Balance between birthing women and organization transformed to balance between midwifery and organization

##### Balance between birthing women and organization (pre-intervention)

A pre-intervention finding was that midwives experienced that their professional role involved balancing the women’s needs and the organization’s demands. The midwives strived to use a women-centred approach, fulfiling the individual needs and empower the woman to give birth. One midwife expressed this approach as follows:

Woman-centred care is central in midwifery. We always use this approach, [as] it makes everything work.^(FG1)^

The professional supportive role was important, manifested as physical presence and psychological support in and outside the labour room, making the woman feels safe and secure.

Guidelines, memoranda, and teamwork with other professions controlled the midwifery work, forcing midwives to balance the need of the birthing woman with the demands of the organization.

You also have to relate to the existing rules and regulations.^(FG1)^

Teamwork was both positive and challenging due to different perspectives on birthing and on the care. Midwives described themselves as experts in the field of normal birth and wanted to be in charge until they sought assistance from the obstetricians if anomalies occurred. At the same time, they wanted continuous support from the assistant nurse and did not see the benefits from polarized working conditions.

##### Balance between midwifery and organization (post-intervention)

Post the intervention labour was still seen as a natural process, but midwives expressed the complexity of birthing in new ways. The model was described as making it easier to see the whole picture and identifying what might interact with the process of birth, i.e., a midwifery normal birth perspective. Although the demands from the organization were still present making the midwives poise the different claims.

… the model made you think more … about what may interfere with her birth … Before, you’ve probably thought of what group she belonged to^(FG2)^

The model helped midwives avoid elements that interfered with the art of midwifery and the philosophy of normal birth; instead of focusing mainly on progress in centimetres, interpretation of the foetal heart rate, or medical anomalies they emphasized the whole context of the childbirth. The model was a tool to clarify difficult internal processes like midwives’ intuition and made the midwives work more distinct.

The organization of teamwork was still considered as difficult and midwives stated that the assistant nurses were feeling excluded when working with MiMo. The professional goal of being present and supportive was in part easier to achieve due to a more accepting approach from the team around the birthing woman post-intervention.

#### Midwives—diverse as both profession and person transformed to midwives—an adaptable profession

##### Midwives—diverse as both profession and person (pre-intervention)

One problem with the professional role was the diversity of individual midwives’ way of working. Midwives developed different personality-related working styles in the labour room. For example, some midwives stated that they worked physically close to the woman and others focused on affirmation on a more mental level.

Because everyone is unique … it is person bound how you work … ^(FG1)^

These dissimilarities could influence the atmosphere when a new midwife entered the labour room in both positive and negative ways. However, the differences were mainly seen as strengths rather than disadvantages. After recognizing their limitations, it was seen as important to find new ways of working around the limitations to fulfil the demands of midwifery work. However, it was up to every individual midwife to deal with the different situations and how it was solved depended to a large extent on how confident they were as a person and as a midwife.

It is very much up to me as a midwife, how confident I am in some situations^(FG1)^

##### Midwives—an adaptable profession (post-intervention)

Midwives reflected post-intervention on their role in the labour room, their working methods and the factors that affected the birthplace. Post-intervention midwives emphasized that they as a profession were adaptable and accommodated their work to the individual woman. Pre the intervention the focus was on the diversity of midwives both as professionals and individuals.

I make different assessments ….based on this situation, this moment … ^(FG3)^

They perceived their work as independent and that it was influenced by their previous knowledge. It was important to be aware of one’s attitudes, norms, and values to accommodate the woman’s needs.

… you reflect all the time on what to change^(FG2)^

Midwives made different decisions depending on the situation in an exact moment, taking into account the woman’s needs, the whole context, and midwifery knowledge.

#### Strained work situation (pre- and post-intervention)

Midwives work situation and professional role were both pre and post the intervention described as influenced by a strained work situation. Midwives assumed full responsibility and commitment to the birthing process. Birthing was seen as the most significant life event for the woman and her partner, but to contribute to that experience was at times sacrificed due to a heavy workload. In these situations, midwives reported that they felt insufficient and torn between different women despite knowing that the best thing would have been one-to-one care. Midwives expressed a self-criticism connected to their high standard goal of care, where they felt insufficient and guilty of not being good enough. They had a sense of deficiency in their ability to fulfil the needs of all the women giving birth. It was described as challenging to have these high demands on oneself, but it was seen as easier to manage when they had been working as a midwife for some time.

You put in an incredible amount of effort trying to do the best you can … but even then you don’t succeed^(FG1)^

The midwives described a lack of support for the supporter, this, they meant, led to midwives not coping with the demands of midwifery in a constructive manner. Post the intervention there was a better understanding of the need of just being in the birthing room as well as focusing on midwifery, but at the same time, there was still major distress regarding the sense of inadequacy. Staff turnover hampered the ability to get guidance and passing on midwifery knowledge, which was experienced as affecting the work situation and professional role negatively.

To be able to give support to the woman, I need to be supported^(FG3)^

Pre and post the intervention the labour ward was described as a stressful environment with personal and complex encounters. Lack of time from the first meeting with the couple through the whole labour process and postpartum was emphasized. The first important encounter had to be hastened resembling speed dating rather than a real encounter to build a trustful relationship.

Midwives knew how they wanted to work connected to MiMo, but they had to prioritize another way to work. It was easy to get caught in the throughput of women giving birth and feel the pull from many directions. Time pressure led to midwifery handcraft being neglected for the benefit of the most acute events. The midwives described an overuse of oxytocin to hasten the birthing process even though the midwives knew it was not necessary. However, midwives expressed that they did not have time to support the woman or wait for the natural process or even reflect on why the oxytocin was administrated.

When the pressure is this high, midwifery skills are set aside, even though you know that it [midwifery skills] is needed and makes everything better … ^(FG2)^

MiMo was seen as a support during heavy workload, enabling the midwives to focus on the most important aspects of midwifery. On the other hand, it also made it clear that they were not able to achieve every goal of midwifery due to the strained work situation.

#### Ability to concretize midwifery a new category post-intervention

Post the intervention the participants had reached a clarity in the concretization of midwifery and evoked a desire to find deeper aspects of the profession. The model granted midwives with conceivable phrases to articulate the concealed or intuitional parts of the profession and gained access to grounded midwifery knowledge.

When you have this midwifery knowledge … you have words for what you previously did more intuitively.^(FG2)^

Pre the intervention reflection was not prioritized and was even disparaged in the work situation that prevailed in the labour ward. Midwives saw MiMo and the professional reflection as a way to visualize midwifery and to develop midwifery knowledge and support one another. It was essential to have time to reflect on what one was doing and feeling while doing emotionally and physically draining work.

We have a great need to reflect on our profession^(FG3)^

To communicate midwife to midwife was not always simple because both criticism and appraisal might be perceived as connected to the person rather than the action. There was little to no time to communicate undisturbed, which led to fragmented conversations.

The basic midwifery concepts used in MiMo were seen as promoting a reflective perspective on the midwifery professional role.

### Quantitative supplemental component results

The quantitative analyses were driven by the qualitative theoretical results—i.e., the survey was reviewed section by section to identify the items that had measurements corresponding to the qualitative categories (Table IV).

There were no significant differences between pre and post measurements for Burnout, Stress, Demand and Control, Organization Climate, and Sense of Coherence, indicating that the MiMo intervention did not affect the measured factors.

### Synthesized results

Synthesizing the qualitative pre-post analyses revealed both corresponding and contrasting results and they are presented in the qualitative core component result and in Table III. The synthesized result of the qualitative and quantitative components showed corresponding and contrasting results in the sections that had representation in the quantitative data (Figure 3).

Post the intervention, the focus had shifted in the qualitative results from Balance between Birthing woman and organization to Balance between Midwifery and organization, leading to contrasting results in QUAL pre-post. MiMo led to midwifery being more prominent in relation to the organization and profession rather than the focus being merely on the individual woman without a midwifery and organizational perspective. This post-intervention change was not seen in the quantitative results. There were no significant differences over time in social support, work ability, worries, or organizational climate. Thus, it was a contrasting result between qualitative and quantitative due to a change in the qualitative categories pre-post and no significant difference in the quantitative result.

There was also a change in focus in the qualitative categories *Midwives—Diverse as both Profession and Person* pre-intervention and *Midwives—An Adaptable Profession* that worked even more woman-centred, and according to the philosophy of normal birth midwifery, post intervention. Consequently, contrasting result in QUAL where MiMo constitutes a more conceptualized collective view of the profession. These categories had no representation in the quantitative analyses.

A new contrasting category emerged post the intervention, *Ability to concretize midwifery*. MiMo enabled the midwives to structure and concretize midwifery and bestowed them with a language to express the grounded midwifery knowledge and the more intuitive and hidden part of the profession. *Ability to concretize midwifery* had no representation in the quantitative data, which mainly focused on the work situation rather than the profession.

The *Strained work situation* was perceived at pre- and post-intervention in both the qualitative and the quantitative results. As stated before, there were no significant differences in the quantitative measurements, which indicate that working according to MiMo did not have any effect on the strained work situation in the context and with the measurements studied here. The overall view of the measurements and interviews revealed that midwives worked in a strained context with a lack of support for the supporter both before and after the MiMo intervention. Despite the strained context midwives reported a strong sense of coherence. Midwives also expressed high demands on themselves as well as a strong commitment to the woman in their care.

The synthesized pre-intervention findings showed that midwives had an individualized understanding of their professional role and their work. The post-intervention findings showed a developed understanding of the contextual impact as well as MiMo having a potential to strengthening midwifery practice and the professional role. However, MiMo appears not to have any measured effect on the midwives’ strained work situation and lack of support for the supporter, in the context and with the measurements studied here.

### Discussion

The aim of this study was to explore and analyse the experience of work situation and professional role for midwives at a labour ward pre and post the implementation of MiMo. The findings from this study showed that post-MiMo midwives expressed a change from a balance between woman and organization to a balance between midwifery and organization, and that they developed an awareness of the contextual impact on their work and professional role, as well as an ability to concretize midwifery. However, the reported strained work situation was evident both pre- and post-intervention.

A qualitative study in the MiMo project shows similar findings. From expressing no need of a midwifery model of care, midwives, obstetricians, and managers expressed that it gave words to woman-centred midwifery care. Further, there was a need for clarifications of professional roles and interdisciplinary collaboration (Lundgren et al., 2020). However, according to the study presented here the strained work situation and lack of support for the supporter did not change after the intervention. On the one hand, MiMo was seen as a tool for support, enabling the midwives to focus on the important aspects of midwifery as an adaptable profession as well as giving them the ability to structure and concretize midwifery in relation to the birthing woman, the other professions, and the organization. On the other hand, MiMo also made it clear to the midwives that they were not able to achieve every goal of midwifery due to the strained work situation and lack of support for the supporter. A study from 2019 by (Geraghty et al., 2019) found that midwives were unable to “do” midwifery in their preferred way, and their work performance depended on the contextual and environmental factors rather than the actual job. The new evident awareness of midwifery, and how the midwives wanted to work, was in contrast to the strained work situation and the medical focus that prevailed in the birth clinic. That dissonance may help explain why the strained work situation did not change after MiMo. The findings in this study must be related to the context: MiMo was implemented in a hospital setting where conventional care followed the medical technocratic model (Davis-Floyd, 2001), and midwifery-led care or continuity care models, providing continuous support during childbirth (Sandall et al., 2016), were not offered.

The strained work situation on all levels (individual, group, and organization) did not change over time. According to our results, this lack of change could be explained by the general lack of time and deficient support for the supporter (the midwives). There seemed to be an imbalance between job demands and job resources causing a strained work situation for the midwives. In the long term, this could lead to a negative impact on the organizational and medical outcomes (Bakker & Demerouti, 2007; Sandall et al., 2016). One important concept to study further in coming research as well as health-promoting factors and resources in the environmental context (Antonovsky, 1987) and in the interprofessional team around the woman (Hansson et al., 2019). The strained work situation and the context where midwives work was in a Swedish study described by other professions in the same setting as a baby factory with an assembly line principle, which caused a tension between midwives and the organization as well as with other professions (Hansson et al., 2019). This could be another explanation of why MiMo did not change the work situation. A Swedish cross-sectional population study reported that the most common work-related stresses in women, in general, were due to work interfering with leisure time, low influence at work, individual demands and commitment, and indistinct organization and conflicts (Holmgren, Dahlin-Ivanoff et al., 2009). Pezaro et al. (2016) state that the midwifery population, internationally, experience work-related distress from both organizational and occupational sources.

Another Swedish study reported that many nurses and midwives were dissatisfied with their work situation and reported a high level of work-related exhaustion (Gardulf et al., 2008), which in turn affected their quality of life. A strong SOC is associated with perceived good quality of life and good health, especially mental health (Eriksson & Lindström, 2007). Factors contributing to strengthening health even during difficult conditions, such as a strained work situation, are called Generalized Resistance Resources (GRR). GRR can be biological, materialistic, and psychosocial (Antonovsky, 1996; Bauer et al., 2006; Lindström & Eriksson, 2005). People that have sufficient GRR and know how to utilize them have a foundation for developing a strong sense of coherence (SOC), which means they are able to perceive life as comprehensive, meaningful, and manageable (Antonovsky, 1996, 2005) despite a strained work situation. A recent Hungarian study (Gebriné et al., 2019) found that a strong SOC had a positive impact on health and stress among hospital working midwives.

In the present study, midwives reported high demands, a strained work situation, and a lack of support for the supporter that did not change after MiMo. It is known that women in “high demand and low control” working conditions have a higher risk of burnout (Norlund et al., 2010), which is applicable to midwifery work. An Australian study showed that 60% of the midwives at two public hospitals experienced moderate to high levels of emotional exhaustion (Mollart et al., 2013). A Swedish study from 2013 showed that a random sample of midwives had a level of 15% work-related burnout and that about 33% had considered leaving the profession (Hildingsson et al., 2013). In a Danish study comparing midwives with other employees in the service sectors, midwives had the highest levels of both work and personal burnout and second highest on client-related burnout after prison guards. The midwives also reported the highest demands from their clients by all participants in the study (Borritz et al., 2006). These findings are alarming considering that depression, anxiety, and stress are associated with burnout (Creedy et al., 2017).

Working according to midwifery continuity of care models has shown to decrease burnout scores and to increase professional satisfaction as well as being the best practice for women, midwives, and organizations (Homer, 2016). Our synthesized results show that the implementation of a theoretically and clinically well-grounded model did not seem to change the strained work situation even though the results clearly indicate there is a strained work situation for midwives. One explanation could be that the strained work situation was due to lack of resources as well as the medical organization of care, a situation MiMo could not change. Previous research shows that insufficient work resources, lack of staff, shift work, and poor organization were considered the main causes of stress for hospital midwives (Knezevic et al., 2011). It is important to consider the organizational context and work situation in which new models are implemented to secure the occupational health of the professionals targeted.

MiMo was not intended to create less job strain but rather to increase the quality of care and strengthen the midwife’s professional role. However, the hypothesis was that the model could affect the work situation. This study is one of the first to explore the effect of a model not only on the professionals’ experience of the model itself but also on the professionals’ experience of the model in relation to their work situation with a parallel quantitative longitudinal assessment of the work situation. Future studies are needed to give better ground to whether the work situation could be improved by midwifery models of care as well as stating implications for practice. A national study is planned using the results from this and other related research as well as applying a salutogenetic approach to the work situation. An improved occupational health and personal well-being of midwives could be cost-effective for society. This improvement could help retain midwives in birthing care and not impoverish the midwifery profession and knowledge that is required for a sustainable salutogenetic workplace and woman-centred care.

### Method discussion

One weakness is that the model was tested in only one hospital and on a low number of midwives; however, it was a total sample of midwives at that ward. The transferability of the result may have to be explored by further research in other geographic or cultural contexts. Another limitation might be the short time frame of the implementation of MiMo, of just 1 year.

The inductive and deductive qualitative approach is according to (Elo & Kyngäs, 2008) useful when comparing categories at different time points, as is done in this paper. A secondary analysis was used on the rich MiMo interviews in this study, to maximize data utility and to apply a work situation and professional role focus on existing data (Heaton, 2004). However, a limitation is that the interviews were based on another but similar research question than in this study. The interviews, however, contained substantial data in relation to the objectives of the present study. The trustworthiness and credibility of the qualitative results were ensured through constant comparison between the first author (MH) and the co-authors during the analytical process, as well as through quotations presented in the qualitative result section. A purposive sampling was used for the interviews for variation in work experience, and this was achieved with a range between 2 and 33 years throughout the three focus groups. This sampling could facilitate transferability.

The quantitative data were the original data collected for this study. The result of this study could be used as a base for further research were additional interviews and surveys will be performed. The sample size in the quantitative part was low and might have contributed to a difficulty to identify changes. This may impact the generalizability of the quantitative results in this study. Hence, the quantitative results need to be interpreted with caution. One other limitation is the narrow timeframe for the implementation, where a longer period might have affected the work situation in a more effective way.

The dropout in the quantitative part of this study might be a methodological problem due to the possibility of the non-responders being the individuals with the worst perceived work situation post the intervention. This dropout bias might have contributed to the non-significant change over time in the quantitative results. But the non-responders did not differ from the responders regarding measurements pre-intervention. Thus, the drop-out might not have affected the results after all. The qualitatively driven mixed method was used to explore how midwives experienced and described their work situation and professional role in the labour ward and to determine if there were items missing in the surveys. Although the synthesized results showed that midwives had a strained work situation and that it is important to emphasize the professional role and the ability to concretize midwifery. Therefore, the mixed method achieved both a broader and a more in-depth result than a single design would have done. The mixed-method design enriched the understanding of the complexity and multifaceted issue of the work situation in a labour ward.

### Conclusions and clinical implications

The synthesized findings based on the qualitative part show that MiMo has a potential to strengthen the professional role and midwifery practice. As such, MiMo has the capability to offer benefits to the labour wards with additional considerations. However, MiMo appears not to have any effect on the strained work situation in neither the qualitative nor the quantitative result. Improved occupational health and personal well-being of midwives could be cost-effective for society. This improvement could be a key factor to enable retaining midwives in birthing care. As well as optimize midwives job satisfaction, and not impoverish the midwifery profession and knowledge that is required for a sustainable woman-centred care.

## References

[cit0001] Antonovsky, A. (1987). *Unraveling the mystery of health: How people manage stress and stay well* (1 ed.). Jossey-Bass.

[cit0002] Antonovsky, A. (1996). The salutogenic model as a theory to guide health promotion. Health Promotion International, 11(1), 11–14. 10.1093/heapro/11.1.11

[cit0003] Antonovsky, A. (2005). *Hälsans mysterium* (2. utg./förord av Lennart Levi ed.). Natur och kultur.

[cit0004] Bakker, A. B., & Demerouti, E. (2007). The job demands-resources model: State of the art. *Journal of Managerial Psychology*, 22(3), 309–328. 10.1108/02683940710733115

[cit0005] Bauer, G., Davies, J. K., & Pelikan, J. (2006). The EUHPID health development model for the classification of public health indicators. *Health Promotion International*, 21(2), 153–159. 10.1093/heapro/dak002 16401640

[cit0006] Berg, M., Asta Ólafsdóttir, Ó., & Lundgren, I. (2012). A midwifery model of woman-centred childbirth care – In Swedish and Icelandic settings. *Sexual & Reproductive Healthcare*, 3(2), 79–87. 10.1016/j.srhc.2012.03.001 22578755

[cit0007] Borritz, M., Rugulies, R., Bjorner, J. B., Villadsen, E., Mikkelsen, O. A., & Kristensen, T. S. (2006). Burnout among employees in human service work: Design and baseline findings of the PUMA study. *Scandinavian Journal of Public Health*, 34(1), 49–58. 10.1080/14034940510032275 16449044

[cit0008] Bryar, R. M., & Sinclair, M. (2011). *Theory for midwifery practice* (2 ed.). Palgrave.

[cit0009] Cohen, S., Kamarck, T., & Mermelstein, R. (1983). A global measure of perceived stress. *Journal of Health and Social Behavior*, 24(4), 385. 10.2307/2136404 6668417

[cit0010] Creedy, D. K., Sidebotham, M., Gamble, J., Pallant, J., & Fenwick, J. (2017). Prevalence of burnout, depression, anxiety and stress in Australian midwives: A cross-sectional survey. (Report). *BMC Pregnancy and Childbirth*, 17(1), 13. 10.1186/s12884-016-1212-5 PMC5223536 28068942

[cit0011] Curtis, P., Ball, L., & Kirkham, M. (2006). Why do midwives leave? (Not) being the kind of midwife you want to be. *British Journal of Midwifery*, 14(1), 27–31. 10.12968/bjom.2006.14.1.20257

[cit0012] Curtis, P. Ball, L., & Kirkham, M. (2005). Why do midwives leave? (Not) being the kind of midwife you want to be. British Journal of Midwifery, 14(1), 27-31. doi: 10.12968/bjom.2006.14.1.20257.

[cit0013] Davis-Floyd, R. (2001). The technocratic, humanistic, and holistic paradigms of childbirth. *International Journal Of Gynecology & Obstetrics*, *75*(2), S5–S23. 10.1016/S0020-7292(01)00510-0 29645265

[cit0014] Elo, S., & Kyngäs, H. (2008). The qualitative content analysis process. *Journal of Advanced Nursing*, 62(1), 107–115. 10.1111/j.1365-2648.2007.04569.x 18352969

[cit0015] Eriksson, M., & Lindström, B. (2007). Antonovsky’s sense of coherence scale and its relation with quality of life: A systematic review. *Journal of Epidemiology and Community Health*, 61(11), 938. 10.1136/jech.2006.056028 17933950 PMC2465600

[cit0016] Eriksson, M., & Mittelmark, B. M. (2017). *The sense of coherence and its measurement*. 28590637

[cit0017] Fahy, K. (2012). What is woman-centred care and why does it matter? *Women and Birth*, 25(4), 149–151. 10.1016/j.wombi.2012.10.005 23177665

[cit0018] Finlayson, B., Dixon, J., Meadows, S., & Blair, G. (2002). Mind the gap: The extent of the NHS nursing shortage. *BMJ*, 325(7363), 538. 10.1136/bmj.325.7363.538 12217997 PMC1124061

[cit0019] Gardulf, A., Orton, M., Eriksson, L. E., Undén, M., Arnetz, B., Kajermo, K. N., & Nordström, G. (2008). Factors of importance for work satisfaction among nurses in a university hospital in Sweden. *Scandinavian Journal of Caring Sciences*, 22(2), 151–160. 10.1111/j.1471-6712.2007.00504.x 18489684

[cit0020] Gebriné, K. É., Lampek, K., Sárváry, A., Sárváry, A., Takács, P., & Zrínyi, M. (2019). Impact of sense of coherence and work values perception on stress and self-reported health of midwives. *Midwifery*, *77*(10), 9–15. 10.1016/j.midw.2019.06.006 31233991

[cit0021] Geraghty, S., Speelman, C., & Bayes, S. (2019). Fighting a losing battle: Midwives experiences of workplace stress. *Women and Birth: Journal of the Australian College of Midwives*, 32(3), e297–e306. 10.1016/j.wombi.2018.07.012 30082214

[cit0022] Hansson, M., Lundgren, I., Hensing, G., & Carlsson, I.-M. (2019). Veiled midwifery in the baby factory - A grounded theory study. *Women and Birth*, 32(1), 80–86. 10.1016/j.wombi.2018.04.012 29709432

[cit0023] Heaton, J. (2004). *Reworking qualitative data*. SAGE Publications.

[cit0024] Henriksen, L., & Lukasse, M. (2016). Burnout among Norwegian midwives and the contribution of personal and work-related factors: A cross-sectional study. *Sexual & Reproductive Healthcare*, *9*(10), 42–47. 10.1016/j.srhc.2016.08.001 27634664

[cit0025] Hildingsson, I., Westlund, K., & Wiklund, I. (2013). Burnout in Swedish midwives. *Sexual & Reproductive Healthcare*, 4(3), 87–91. 10.1016/j.srhc.2013.07.001 24041728

[cit0026] Holmgren, K., Dahlin-Ivanoff, S., Björkelund, C., & Hensing, G. (2009). The prevalence of work-related stress, and its association with self-perceived health and sick-leave, in a population of employed Swedish women. *BMC Public Health*, 9(1), 73. 10.1186/1471-2458-9-73 19254367 PMC2653036

[cit0027] Holmgren, K., Hensing, G., & Dahlin-Ivanoff, S. (2009). Development of a questionnaire assessing work-related stress in women – Identifying individuals who risk being put on sick leave. *Disability and Rehabilitation*, *31*(4), 284–292. 10.1080/09638280801931287 18720119

[cit0028] Homer, C. S. (2016). Models of maternity care: Evidence for midwifery continuity of care. *Medical Journal of Australia*, 205(8), 370–374. 10.5694/mja16.00844 27736625

[cit0029] Horiuchi, S., Kataoka, Y., Eto, H., Oguro, M., & Mori, T. (2006). The applicability of women‐centered care: Two case studies of capacity‐building for maternal health through international collaboration. *Japan Journal of Nursing Science*, 3(2), 143–150. 10.1111/j.1742-7924.2006.00060.x

[cit0030] Hunter, L. (2016). Making time and space: The impact of mindfulness training on nursing and midwifery practice. A Critical Interpretative Synthesis. *Journal of Clinical Nursing*,*25*(7-8), 918–929. 10.1111/jocn.13164 26748547

[cit0031] Karasek, R. (1990). *Healthy work: Stress, productivity, and the reconstruction of working life*. Basic Books.

[cit0032] Knezevic, B., Milosevic, M., Golubic, R., Belosevic, L., Russo, A., & Mustajbegovic, J. (2011). Work-related stress and work ability among Croatian university hospital midwives. *Midwifery*, 27(2), 146–153. 10.1016/j.midw.2009.04.002 19589631

[cit0033] Kristensen, T. S., Borritz, M., Villadsen, E., & Christensen, K. B. (2005). The copenhagen burnout inventory: A new tool for the assessment of burnout. *Work and Stress*, 19(3), 192–207. 10.1080/02678370500297720

[cit0034] Leap, N. (2009). Woman-centred or women-centred care: Does it matter? *British Journal of Midwifery*, 17(1), 12–16. 10.12968/bjom.2009.17.1.37646

[cit0035] Lindström, B., & Eriksson, M. (2005). Salutogenesis. *Journal of Epidemiology and Community Health*, 59(6), 440. 10.1136/jech.2005.034777 15911636 PMC1757059

[cit0036] Lundgren, I., Berg, M., Nilsson, C., & Olafsdottir, O. A. (2020). Health professionals’ perceptions of a midwifery model of woman-centred care implemented on a hospital labour ward. *Women and Birth, 33*(1), 60–69. 10.1016/j.wombi.2019.01.004 30686654

[cit0037] MacKenzie Bryers, H., & van Teijlingen, E. (2010). Risk, theory, social and medical models: A critical analysis of the concept of risk in maternity care. *Midwifery*, 26(5), 488–496. 10.1016/j.midw.2010.07.003 20719418

[cit0038] Mackin, P., & Sinclair, M. (1998). Labour ward midwives’ perceptions of stress. *Journal of Advanced Nursing*, 27(5), 986–991 986. 10.1046/j.1365-2648.1998.t01-1-00571.x 9637325

[cit0039] Magistretti, C. M., Downe, S., Lindstrøm, B., Berg, M., & Schwarz, K. T. (2016). Setting the stage for health: Salutogenesis in midwifery professional knowledge in three European countries. *International Journal of Qualitative Studies on Health and Well-being*, 11(1), 33155. 10.3402/qhw.v11.33155

[cit0040] Mollart, L., Skinner, V. M., Newing, C., & Foureur, M. (2013). Factors that may influence midwives work-related stress and burnout. *Women and Birth*, 26(1), 26–32. 10.1016/j.wombi.2011.08.002 21889431

[cit0041] Morse, J. M. (2016). *Mixed method design: Principles and procedures*. Taylor & Francis.

[cit0042] Morse, J. M., & Niehaus, L. (2009). *Mixed method design: Principles and procedures*. Left Coast Press.

[cit0043] Norlund, S., Reuterwall, C., Höög, J., Lindahl, B., Janlert, U., & Birgander, L. S. (2010). Burnout, working conditions and gender - results from the northern Sweden MONICA Study. *BMC Public Health*, 10(1), 326. 10.1186/1471-2458-10-326 20534136 PMC2896942

[cit0044] Olafsdottir,O.A., 2006. An Icelandic midwifery saga—coming to light with women and connective ways of knowing. Doctoral Dissertation, Thames Valley University. London.

[cit0045] Pezaro, S., Clyne, W., Turner, A., Fulton, E. A., & Gerada, C. (2016). ‘Midwives Overboard!’ Inside their hearts are breaking, their makeup may be flaking but their smile still stays on. *Women and Birth,29*(3),59-66. 10.1016/j.wombi.2015.10.006 26522961

[cit0046] Sandall, J., Soltani, H., Gates, S., Shennan, A., & Devane, D. (2016). Midwife-led continuity models versus other models of care for childbearing women. *Cochrane Database Of Systematic Reviews*, 4(4). 10.1002/14651858.CD004667.pub5 PMC8663203 27121907

[cit0047] Schoonenboom, J., & Johnson, R. B. (2017). How to construct a mixed methods research design. *Kolner Zeitschrift Fur Soziologie Und Sozialpsychologie*, 69(Suppl 2), 107–131. 10.1007/s11577-017-0454-1 28989188 PMC5602001

[cit0048] Zhang, J., Haycock-Stuart, E., Mander, R., & Hamilton, L. (2015). Navigating the self in maternity care: How Chinese midwives work on their professional identity in hospital setting. *Midwifery*, 31(3), 388–394. 10.1016/j.midw.2014.11.013 25533150

